# Antibacterial activity and mechanism of Sodium houttuyfonate against heteroresistant *Pseudomonas aeruginosa*

**DOI:** 10.3389/fmicb.2025.1523182

**Published:** 2025-03-06

**Authors:** Zhihong Li, Tongtong Zhang, Ziqi Wang, Shuqiang Huang, Cuiyu Tan, Dan Wang, Xiaojun Yuan, Lingqing Xu

**Affiliations:** ^1^Department of Laboratory, The Affiliated Qingyuan Hospital (Qingyuan People's Hospital), Guangzhou Medical University, Qingyuan, China; ^2^Department of Clinical Laboratory, The First People’s Hospital of Foshan, Foshan, China

**Keywords:** *Pseudomonas aeruginosa*, heteroresistant *Pseudomonas aeruginosa*, Meropenem, Sodium houttuyfonate, biofilm

## Abstract

**Background:**

*Pseudomonas aeruginosa* is a common gram-negative opportunistic pathogen that is now commonly treated with carbapenems, such as Meropenem. However, the increasing rate of emergence of heteroresistant strains poses a therapeutic challenge. Therefore, we examined the antibacterial activity of Sodium houttuyfonate (SH, a compound derived from *Houttuynia cordata*) in combination with Meropenem (MEM) against heteroresistant *Pseudomonas aeruginosa* and investigated the mechanism of Sodium houttuyfonate.

**Methods:**

Heteroresistant *Pseudomonas aeruginosa* was used as the experimental strain for the study and the combined action activity of the two drugs was inves-tigated by determining the Minimum Inhibitory Concentration (MIC), Fractional Inhibitory Concentration Index (FICI), and time killing curves. Also the effect of Sodium houttuyfonate on biofilm as well as bacterial swimming motility assay was investigated by crystal violet staining of bacterial biofilm, microanalysis of biofilm, bacterial swimming motility assay, quantitative real-time PCR (qRT-PCR) and population sensing related virulence factors.

**Results:**

For the screened experimental strains, the MIC of SH was 4,000 μg/ml; the FICI of both drugs on the four experimental strains was ≤0.5, which showed a synergistic effect. When SH ≥ 250 μg/ml, it was able to effectively inhibit bacterial biofilm formation as well as swimming ability compared with the blank control group. In the qRT-PCR experiment, the expression of biofilm formation-related genes (*pslA*, *pelA*, *aglD*, *lasI*, *lasR*, and *rhlA*) and swimming ability-related genes (*fliC*, *pilZ*, and *pilA*) were decreased in the SH-treated group, compared with the blank control group.

**Conclusion:**

Our study demonstrated that Sodium houttuyfonate and Meropenem exhibited synergistic inhibition against heteroresistant *Pseudomonas aeruginosa*, and that Sodium houttuyfonate may achieve its inhibitory effect by inhibiting bacterial biofilm formation, inhibiting motility, and down-regulating related genes.

## 1 Introduction

*Pseudomonas aeruginosa*, a Gram-negative bacterium, is widely distributed on the human body surface and in the intestinal tract and is a common opportunistic pathogen. *Pseudomonas aeruginosa* is one of the main causative agents of nosocomial infections and can cause infections in different parts of the body in immunocompromised patients, which may lead to wound and skin infections, pneumonia, bacteraemia, sepsis (Buehrle et al., 2017).

In general terms, heteroresistance to antibiotics mainly refers to the presence in clinical isolates of one or several subgroups of drug-resistant bacteria that, unlike the majority of their homologues, have a significantly reduced susceptibility to antibiotics (El-Halfawy and Valvano, 2015). Heteroresistant to multiple antibiotics is now found to be common in clinical isolates of *Pseudomonas aeruginosa*, which may be an important risk factor for failure of clinical anti-infective therapy as well as bacterial resistance (El-Halfawy and Valvano, 2015). Although heteroresistant strains of *Pseudomonas aeruginosa* have been reported for many years, the mechanism of heteroresistant in *Pseudomonas aeruginosa* remains unclear.

Meropenem is a carbapenem drug commonly used clinically against *Pseudomonas aeruginosa* (Pulmonary Infection Assembly of Chinese Thoracic, 2022). It has been noted (Bubonja-Sonje et al., 2015) that the resistance of *Pseudomonas aeruginosa* to Meropenem is mainly achieved through the exocytosis pump and down-regulation of the oprD gene. Research has shown that with the increased frequency of antibiotic use in recent years, there is a high proportion of multi-drug resistant *Pseudomonas aeruginosa* in hospital-acquired pneumonia, with the proportion of carbapenem-resistant *Pseudomonas aeruginosa* being as high as 36.6 to 44.8% (Yin et al., 2021). Yang Lu (Lu et al., 2022) investigated the rate of heteroresistant to different antibiotics among clinical *Pseudomonas aeruginosa* isolates, and 58.2% of the 170 strains were heterogeneously resistant to Meropenem.

*Houttuynia cordata* is a perennial plant, mostly distributed in East Asian countries (Wu et al., 2021). In China, *Houttuynia cordata* has long been used as food and medicine. In traditional Chinese medicine, it’s generally used to treat inflammation, rheumatoid arthritis, viral or bacterial infections, hyperglycaemia and other diseases (Kumar et al., 2014). In recent years, more and more studies have shown that *Houttuynia cordata* has good antimicrobial activity. Sodium houttuyfonate, as one of the main components of *Houttuynia cordata*, and its compounds with *Houttuynia cordata* are inhibitory to *Pseudomonas aeruginosa* (Laldinsangi, 2022). Wu et al. (2015) showed that Sodium houttuyfonate significantly inhibited *Pseudomonas aeruginosa* biofilm formation by decreasing the expression of alginate, the main component of biofilm, and also decreased the motility of *P. aeruginosa*. He also stated in another study that Sodium houttuyfonate can inhibit *Pseudomonas aeruginosa* virulence and resistance by effectively inhibiting the systems regulated by quorum sensing (Wu et al., 2014).

The present study was conducted to explore the effect of Sodium houttuyfonate combined with Meropenem on the antibacterial activity of heteroresistant *Pseudomonas aeruginosa* and its mechanism using several heteroresistant *Pseudomonas aeruginosa* strains isolated from the clinic. This study investigated the *in vitro* antibacterial efficacy of sodium houttuyfonate against *Pseudomonas aeruginosa*, aiming to provide experimental evidence for its potential therapeutic application in combating *Pseudomonas aeruginosa* infections.

## 2 Materials and methods

### 2.1 Experimental strains

All the strains in this experiment were obtained from the strain preservation library of the Microbiology Department of the Laboratory of Qingyuan People’s Hospital. Twenty strains of extensively drug-resistant *Pseudomonas aeruginosa* were randomly selected from the strain preservation bank, and the specimens were taken from blood, sputum, pleural and abdominal fluid and other fluids of clinical patients in our hospital.

### 2.2 Reagents and instruments

Sodium houttuyfonate (HARVEYBIO, 97%, high purity); Meropenem (Solarbio/Solarbio); Meropenem drug-sensitive paper tablets (Liofilchem/Liofilchem); sterile PBS solution (ECOTOP SCIENTIFIC); blood plate (Jiangmen Kailin Trading Co., Ltd.); broth medium and MH agar plate (Guangzhou Dijing Ltd.); LB liquid medium (tryptone 10 g/L, yeast extract 5 g/L, sodium chloride 10 g/L); 1% crystal violet staining solution (Solarbio/Solarbio); Trizol lysate (BioFluX); reverse transcription kit (MedChem Express); SYBR Green qPCR Master Mix (MedChem Express); 96-well plate (Beijing Xinxing Qiangsen Biotechnology Co, Ltd.); DensiCHEK Plus Electronic Turbidimeter (bioMérieux USA Inc.); essenscienELF6 mini centrifuge (Guangzhou Keqiao Experimental Technology Equipment Co, Ltd.); KDC-40 low-speed centrifuge (Anhui Zhongke Zhongjia). (Anhui Zhongke Zhongjia); desktop high-speed freezing centrifuge (Germany SIGMA1-16K); enzyme labelling instrument (Germany BMGSpectrostarNamo full-wavelength); BIOMATE160 UV spectrophotometer (Thermo Fisher Scientific); orthogonal fluorescence microscope (Japan Olympus BX53); fluorescence quantitative PCR (Bohle CFX-CONNEC).

### 2.3 Preparation of bacterial and drug solutions

In order to obtain a single colony, we activated the selected 20 strains of *Pseudomonas aeruginosa*, transferred them to the blood plate by plate streaking method, and incubated them in a microbial incubator at 37 ° C for 24 h. After 24 h of culture, single colonies were picked with a sterile disposable cotton swab, mixed in a sterile PBS solution, and the concentration of the solution was adjusted to 1.5 × 10^8^ CFU/ml. Sodium houttuynium and Meropenem were dissolved in pure water, and the concentration was adjusted to 64,000 μg/ml and 2028 μg/ml, respectively.

### 2.4 Screening for heteroresistant strains

Screening for heteroresistant strains was performed using the Kirby-Bauer disk diffusion test. A disposable sterile cotton swab was used to spread the bacterial solution at a concentration of 1.5 × 10^8^ CFU/ml over the entire surface of the MH medium. After the liquid was absorbed, a Meropenem disk was placed in the centre of the medium and incubated in a microbiological incubator at 37°C for 24 h. We preliminarily recognized the colonies growing in the inhibition circle as heteroresistant strains by visual observation. The strains initially recognized as heterogeneous were identified as homologous to the original strain by the VITER-2 Compact fully automated microbiological analysis system. According to the literature, if Highest Inhibitory Concentration (HIC) at which the antibiotic exhibits the greatest inhibitory effect on the bacteria is higher than eight times the Highest Noninhibitory Concentration (HNIC), then the strain can be defined as a heteroresistant strain (El-Halfawy and Valvano, 2015). Four heteroresistant strains were identified, and were used in the subsequent experiments, with the numbers of strains 10,512, 10,517, 11,617, and 11,643, respectively.

### 2.5 Determination of minimum inhibitory concentration

The MICs of Sodium houttuyfonate and Meropenem against heteroresistant strains of bacteria was determined by micro broth dilution method. To the 96-well plate, 100 μl each of the bacterial solution with 1.5 × 10^8^ CFU/ml and the drug solution with diluted concentration were added, making the final concentration of Sodium houttuyfonate 32,000, 16,000, 8,000, 4,000, 2,000, 1,000, 500, 250, 125, 63, 32, 16 μg/ml; and the final concentration of Meropenem 10,240, 5,120, 2,560, 1,280, 640, 320, 160, 80, 40, 20, 10, 5 μg/ml. sterile LB liquid medium was set as blank control, and the suspension without any drug was negative control, mixed and incubated at constant temperature at 37°C for 20 h. After incubation, the MIC is determined as the lowest concentration of the antimicrobial agent that completely inhibits visible bacterial growth (Parvekar et al., 2020).

### 2.6 Determination of fractional inhibitory concentration index

**Figure 1:**
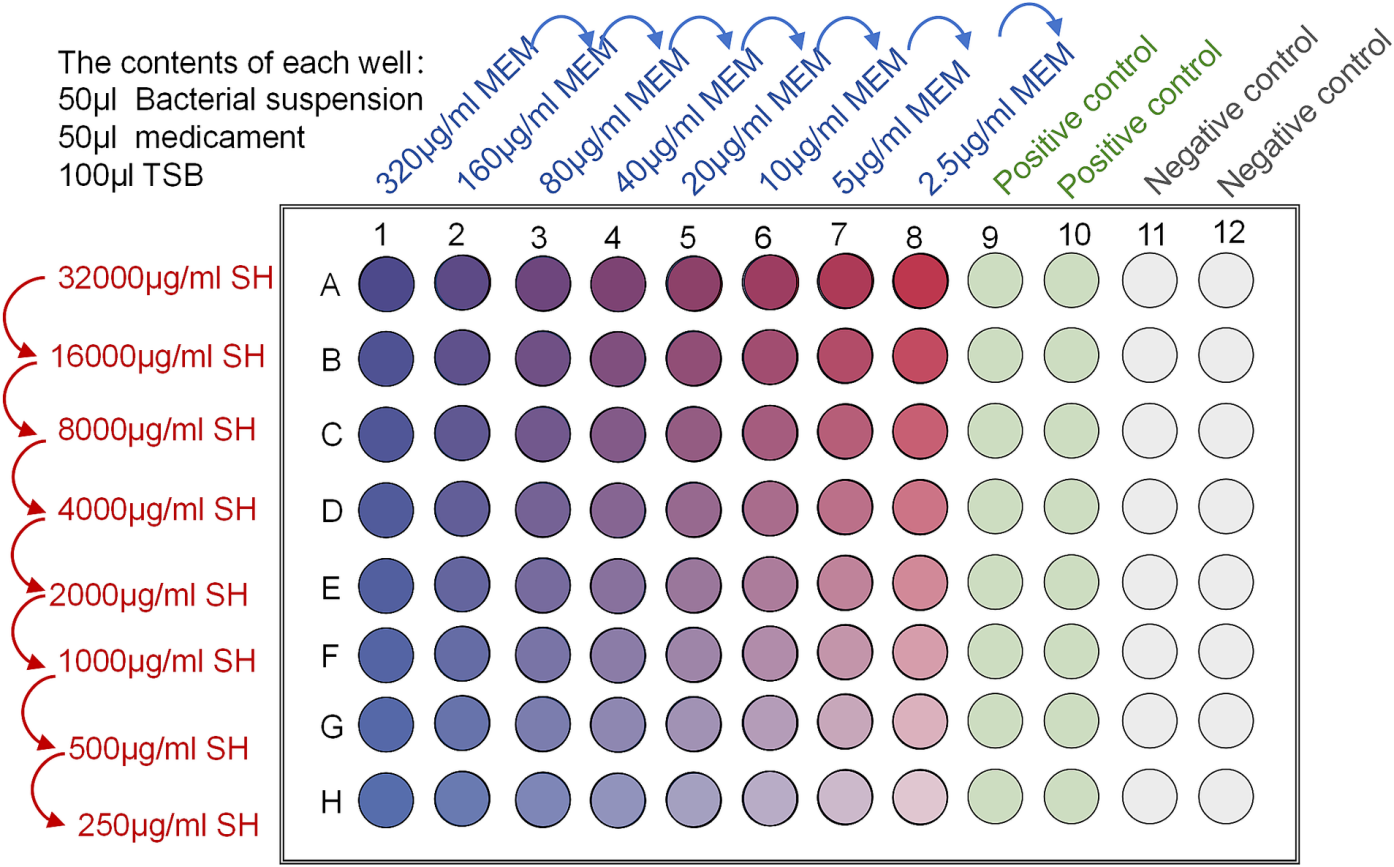
Schematic diagram of FICI determination by checkerboard broth dilution method.

The MICs of Sodium houttuyfonate and Meropenem were measured by the checkerboard dilution method, and the inhibitory index of the combination was calculated to measure the effect of the combination. In the 96-well plate labelled 1–8 horizontal columns, Sodium houttuyfonate was continuously diluted at twice the rate; in the columns labelled A-H, Meropenem was diluted at twice the rate. Add 50 μl of Sodium houttuyfonate and Meropenem into each well; then add 50 μl of 1.5 × 10^8^ CFU/ml bacterial solution into each well, and finally add MH liquid medium into each well to make up to 200 μl. The concentration gradient of Sodium houttuyfonate and Meropenem was determined according to the results of the experiment; meanwhile, set up the sterile LB liquid medium in the columns of 9–12 to be the blank control, and the bacterial solution without any drug was used as the negative control. as a negative control, mixed and incubated at a constant temperature of 37°C for 24 h (Figure 1). After the end of the culture, the OD_600_ of each well was measured by microplate reader, and the MIC of the drug was the smallest concentration of the drug that inhibited more than 80% of the bacteria, FICI = MIC of A drug combination/A drug alone MIC+MIC of B drug combination/B drug alone MIC; FICI≤0.5 is synergistic, 0.5 < FICI≤1 is additive, 1 < FICI≤2 is irrelevant, and FICI > 2 is antagonistic. FICI >2 is antagonistic.

### 2.7 Measurement of time-kill curves

According to the determined MIC values, Sodium houttuyfonate and Meropenem were diluted to 2 times MIC, 1 times MIC, ½ times MIC concentration for each strain with fresh MH liquid medium, respectively. Logarithmically grown bacterial suspension was turbidimetrically adjusted to 1.5 × 10^8^ CFU/ml with MH liquid medium. Each strain was prepared as ① 1 times MIC concentration of SH (1 × MIC SH), ② ½ times MIC concentration of SH (½ × MIC SH), ③ 1 times MIC concentration of MEM (1 × MIC MEM), ④ ½ times MIC concentration of MEM (½ × MIC MEM), ⑤ 1 times MIC concentration of SH and 1 times MIC concentration of MEM (1 × MIC SH + 1 × MIC MEM), ⑥ ½ times MIC concentration of SH and ½ times MIC concentration of MEM (½ × MIC SH + ½ × MIC MEM), and ⑦ negative control; the suspension without any drug was the negative control. Each tube was incubated with the same concentration of the final bacterial solution at 37°C in a constant temperature incubator with shaking; OD_600_ was measured at 8 time points, respectively 0, 4, 8, 12, 24, 36, 48, and 72 h. The time-killing curves of the strains were plotted with the time points as the horizontal coordinates and the OD_600_ as the vertical coordinates (Qu et al., 2016; Rogers et al., 2022).

### 2.8 Experiments on the inhibition of biofilm

The biofilm inhibition test of heterogeneous *Pseudomonas aeruginosa* by Sodium houttuyfonate was determined by Crystal violet staining (Masihzadeh et al., 2023). Suspensions in the logarithmic growth phase were taken and diluted to 1.5 × 10^8^ CFU/ml. LB liquid medium containing different concentrations of Sodium houttuyfonate was prepared using LB liquid medium as a solvent for Sodium houttuyfonate. To each 96-well plate, 100 μl of bacterial suspension and drug solution were added, making the final concentration of Sodium houttuyfonate 125, 250, 500, 750, 1,000, 1,500, 2,000, 4,000, 6,000, and 8,000 μg/ml. each drug concentration possessed three replicate wells, and at the same time, sterile LB liquid medium was set up as a blank control. The suspension without any drug was the negative control. After thorough mixing, the incubation was left at 37°C for 18–24 h. After the incubation, the culture medium was discarded and washed with sterile PBS to preserve the biofilm as much as possible. After drying, the biofilm was fixed with anhydrous ethanol for 15 min; after fixation, the anhydrous ethanol was discarded. The biofilm was stained with 0.1% crystal violet staining solution for 5 min; after staining, the staining solution was washed away with sterile PBS; finally, 33% acetic acid solution was added to dissolve the biofilm after air-drying. The 96-well plate was incubated at 37°C for 30 min, and the absorbance of each well was measured at 570 nm using a microplate reader.

### 2.9 Microtiter plate assay of biofilm inhibition experiments

Put a microscope slide into a 6-well plate, add 0.5 ml of suspension with 1.5 × 10^8^ CFU/ml, 0.5 ml of different concentrations of Sodium houttuyfonate, 1 ml of LB liquid medium and mix thoroughly, so that the final concentrations of Sodium houttuyfonate were 0, 250, 500, 1,000, 2,000 and 4,000 μg/ml. At the same time, set up a negative control group without the addition of Sodium houttuyfonate, and put it into a 37°C incubator for 18–24 h (Haney et al., 2021). The subsequent steps were the same as in experiment 2.8, and the stained coverslips were placed on the slides and observed using a light microscope with a magnification of 1,000 times.

### 2.10 Bacterial swimming motility assay

Bacterial swimming motility assay was used to determine the effect of Sodium houttuyfonate on the swimming ability of heterogeneous *Pseudomonas aeruginosa* (Qu et al., 2016). Swimming plates (LB agar with 0.3% w/v agar) containing different concentrations of Sodium houttuyfonate (0, 250, 500, 1,000 and 2,000 μg/ml) were prepared. After the plates were dried, single colonies were inoculated in the centre of the plate using a sterile toothpick and incubated at a constant temperature of 37°C for 24 h. Swimming diameters of each group were determined and compared with the control group to determine the effect on the ability to swim. All experiments were performed in triplicate using the same bacterial strain, and the mean values of the results were calculated and included in the statistical analysis.

### 2.11 Real-time fluorescence quantitative PCR

Each bacterial strain was set up with Sodium houttuyfonate (2,000 μg/ml) treated group and control group without Sodium houttuyfonate treatment. Total RNA was extracted from biofilm-state bacteria using TRIzol reagent, with RNase-free water serving as a blank control. The A260/A280 ratios of all RNA samples ranged between 1.9 and 2.1, confirming high purity. Equal amounts of RNA were reverse-transcribed into cDNA using a reverse transcription kit according to the manufacturer’s protocol, and stored at −20°C for subsequent use.

Real-time fluorescence quantitative PCR (RT-qPCR): The relative expression levels of genes related to biofilm formation and genes related to swimming ability were calculated according to the 2-ΔΔCt formula using SYRB Green staining method, and the homologous untreated strain of *Pseudomonas aeruginosa* treated with sodium ichthyocyanin at 2,000 μg/ml was used as the reference strain, and *16SrRNA* gene was used as the reference gene. Calculations are made by taking the average of 3 replicate holes for each sample. The average of three wells for each sample was taken for calculation, and the RT-qPCR primers required for this experiment were designed using Primer Premier 6.0 (Supplementary Table S1). The reaction system and conditions were set up according to the instructions of the real-time fluorescence quantitative PCR kit, and the specificity of the products was determined by melting curve analysis.

### 2.12 Statistical analysis

Statistical analyses of data are expressed as mean ± standard error. Independent t-tests were used for comparisons between two groups, and one-way ANOVA was used for comparisons between more than two groups. All statistical analyses were performed using GraphPad Prism 9.0 (GraphPad Software Inc., San Diego, CA, USA). All statistical tests were two-sided and *p* < 0.05 was considered statistically significant.

## 3 Results

### 3.1 Screening for heteroresistant *Pseudomonas aeruginosa*

**Figure 2:**
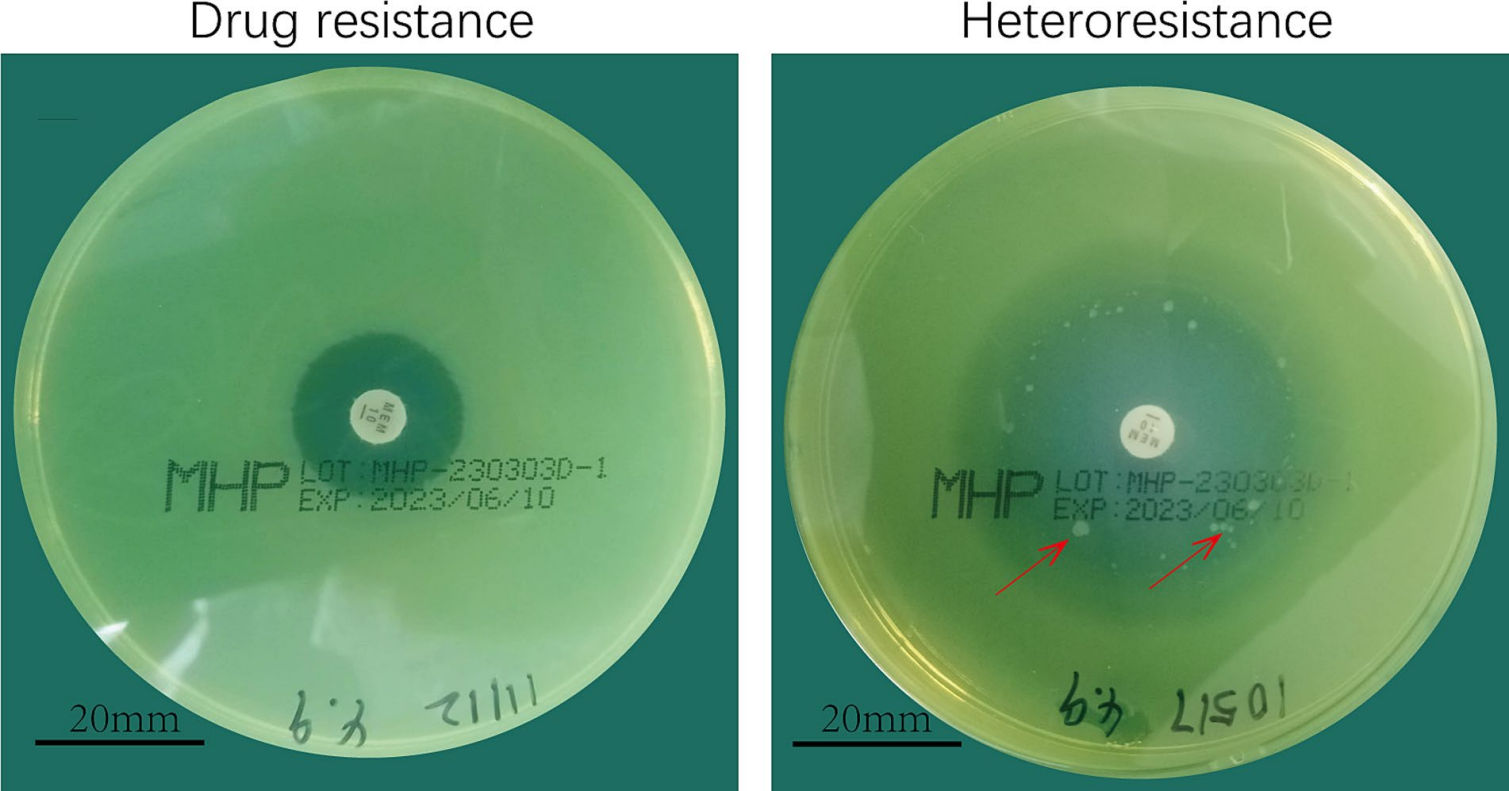
Meropenem heterodrug-resistant *Pseudomonas aeruginosa*. **(A)** Drug-resistant. **(B)** Heteroresistant.

Initial screening using the disk diffusion method revealed scattered colonies within the inhibition zones, suggesting potential heteroresistance (Figure 2). Turbidimetric validation in LB broth confirmed four strains with a heterogeneous inoculum effect (HIC/HNIC ≥8) (Table 1).

**Table 1:** Identification of heteroresistant *Pseudomonas aeruginosa* by Meropenem.

Number	HIC/μg*ml^−1^	HNIC/μg*ml^−1^	NIC/HNIC
10,512	40	1.25	32
11,517	20	0.62	32
11,617	20	1.25	16
11,643	40	1.25	32

When HIC/HNIC ≥ 8, the strain can be judged to be heterogeneously resistant.

### 3.2 Minimum inhibitory concentration

The MICs of Sodium houttuyfonate (SH) and Meropenem (MEM) were determined via broth microdilution in 96-well plates. SH exhibited a consistent MIC of 4,000 μg/ml across all strains, while MEM showed strain-dependent inhibitory activity (Table 2).

**Table 2:** Effects of SH and MEM on experimental strains.

Strain number	SH/μg*ml^−1^	MEM/μg*ml^−1^
10,512	4,000	40
10,517	4,000	20
11,617	4,000	20
11,643	4,000	40

### 3.3 Synergistic effects of SH and MEM

**Figure 3:**
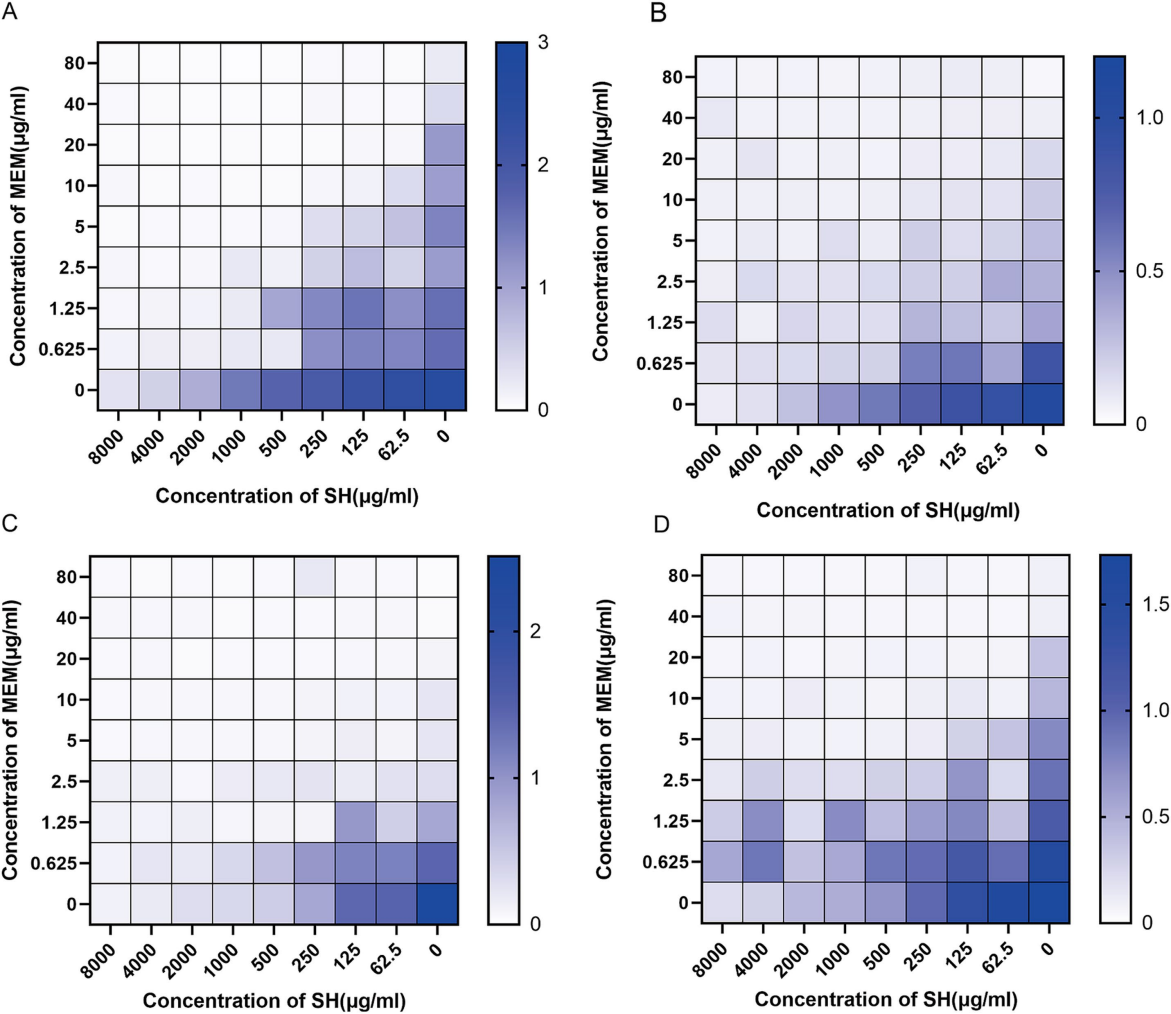
Thermal potency plots of the combined effects of different concentrations of SH and MEM on *Pseudomonas aeruginosa*. **(A)** 10,512. **(B)** 10,517. **(C)** 11,617. **(D)** 11,643.

Checkerboard assays revealed synergistic inhibition (FICI ≤ 0.5) of SH and MEM against all four strains (Table 3). Heatmaps generated with Prism software visualized growth patterns across drug concentration gradients (Figure 3).

**Table 3:** Checkerboard broth dilution assay for the antibacterial effect of the combination of SH and MEM against *Pseudomonas aeruginosa*.

Strain number	MIC for combination therapy/MIC for monotherapy		FICI	Result
	SH	MEM		
10,512	250/4000	2.5/40	0.125	Synergy effect
10,517	125/4000	1.25/20	0.094	Synergy effect
11,617	250/4000	1.25/20	0.125	Synergy effect
11,643	250/4000	2.5/40	0.125	Synergy effect

FICI = MIC of drug A in combination/MIC of drug A alone + MIC of drug B in combination/MIC of drug B alone. FICI ≤ 0.5 was synergistic effect, 0.5 < FICI ≤ 1 was additive effect, 1 < FICI ≤ 2 was irrelevant effect, and FICI > 2 was antagonistic effect.

### 3.4 Time-kill kinetics

**Figure 4:**
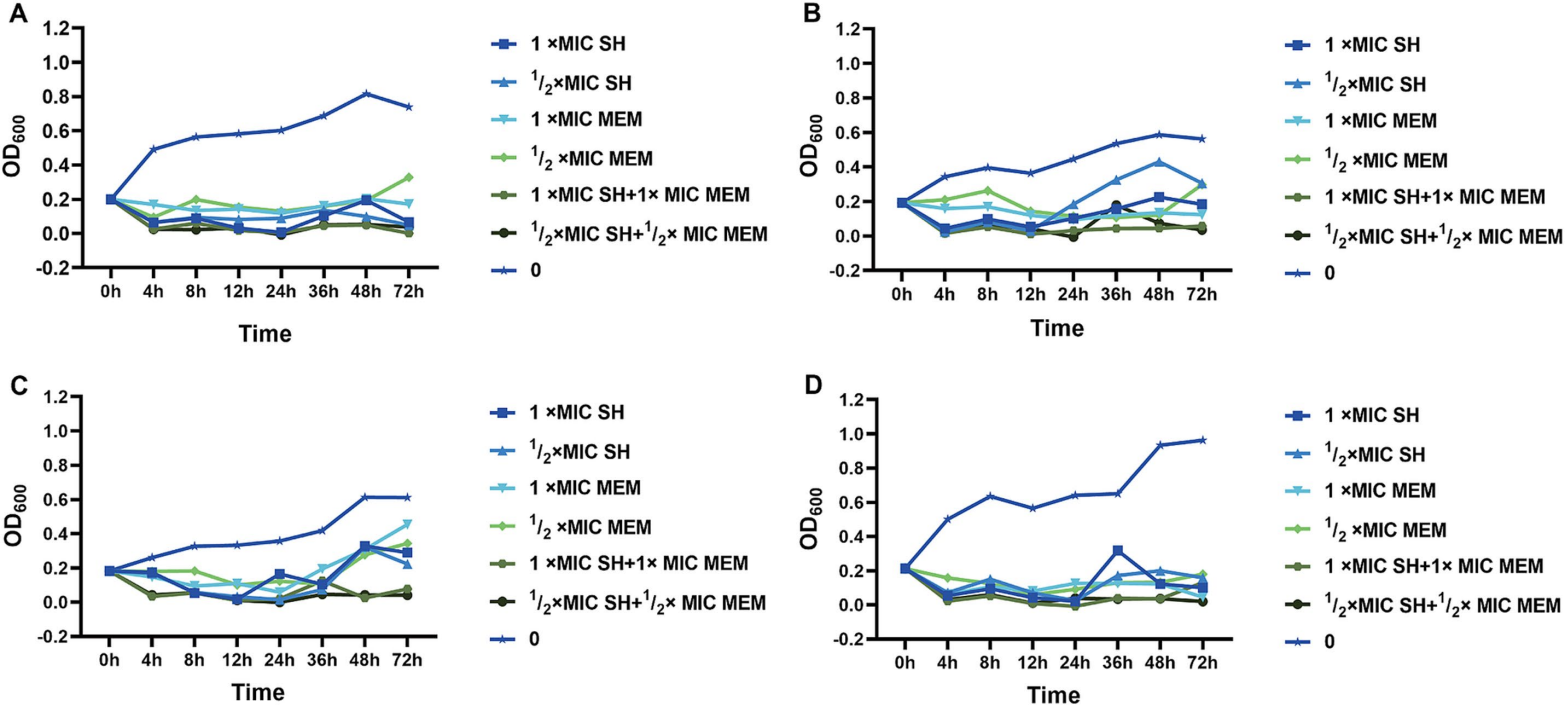
The time bactericidal curve of single and combined action of SH and MEM on strains. **(A)** 10,512. **(B)** 10,517. **(C)** 11,617. **(D)** 11,643.

Time-kill curves demonstrated that SH alone (1 × MIC SH and ½ × MIC SH) achieved bactericidal effects comparable to MEM. However, combinations of SH and MEM (1 × MIC SH + 1 × MIC MEM or ½ × MIC SH + ½ × MIC MEM) exhibited enhanced efficacy, with sustained suppression of bacterial regrowth over 72 h (Figure 4). For example:

- Strain 10,512: Combination therapy reduced viable counts by >1.5 × 10^8^ CFU/ml within 12 h, maintaining suppression until 72 h.
- Strain 11,643: Both 1 × MIC SH + 1 × MIC MEM and ½ × MIC SH + ½ × MIC MEM combinations eradicated bacteria by 4 h, with no regrowth observed.

### 3.5 Biofilm inhibition by SH

**Figure 5:**
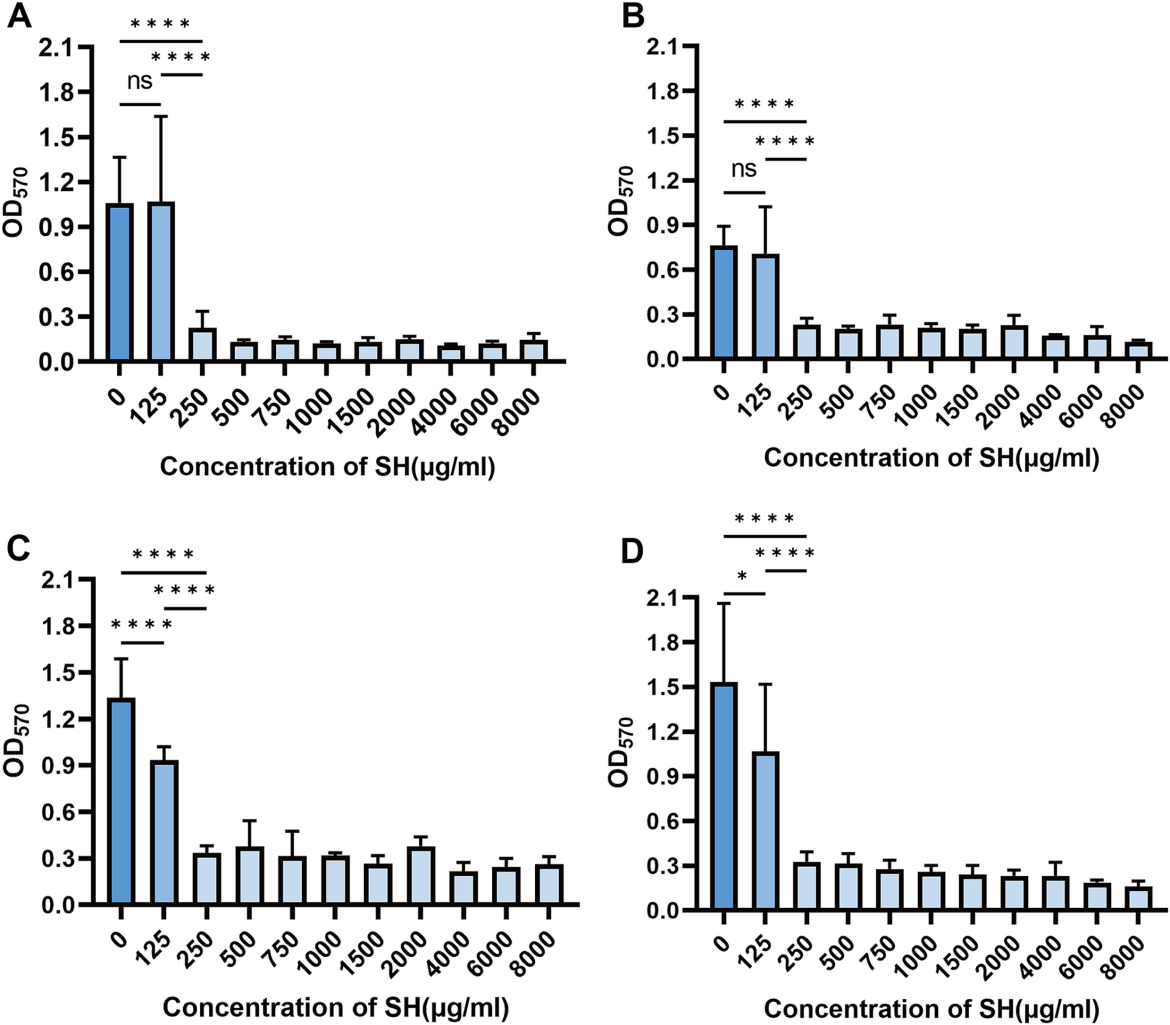
Effect of different concentration of SH on biofilm formation. Where ns denotes no statistical significance, * denotes 0.05 ≤ *p* < 0.1, ** denotes *p* < 0.05, *** denotes *p* < 0.01, and **** denotes *p* < 0.001.Error bars denote SD.**(A)** 10,512. **(B)** 10,517. **(C)** 11,617. **(D)** 11,643.

**Figure 6:**
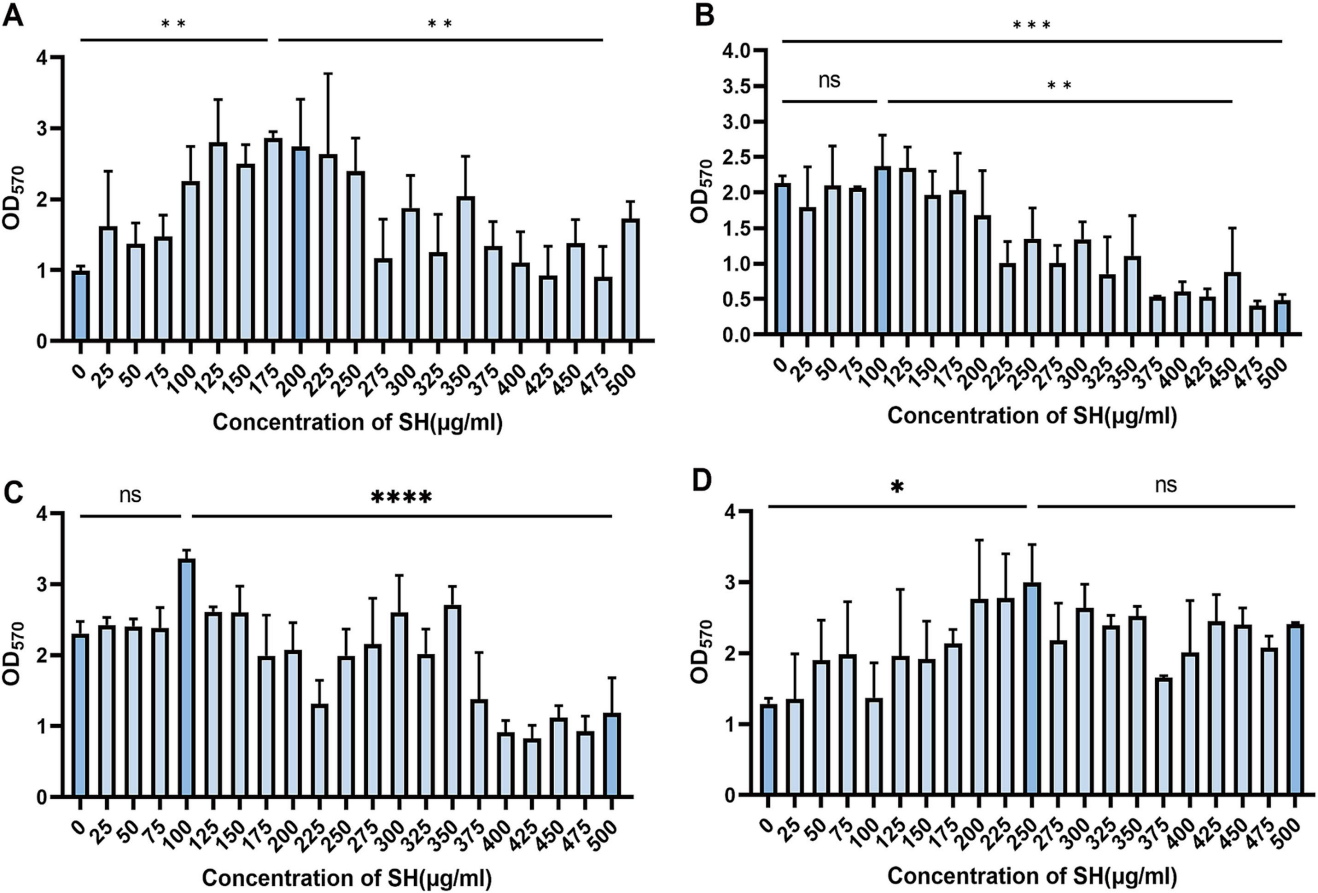
Effect of substatic SH concentration on bacterial biofilm. Where ns denotes no statistical significance, * denotes 0.05 ≤ *p* < 0.1, ** denotes *p* < 0.05, *** denotes *p* < 0.01, and **** denotes *p* < 0.001.Error bars denote SD. **(A)** 10,512. **(B)** 10,517. **(C)** 11,617. **(D)** 11,643.

**Figure 7:**
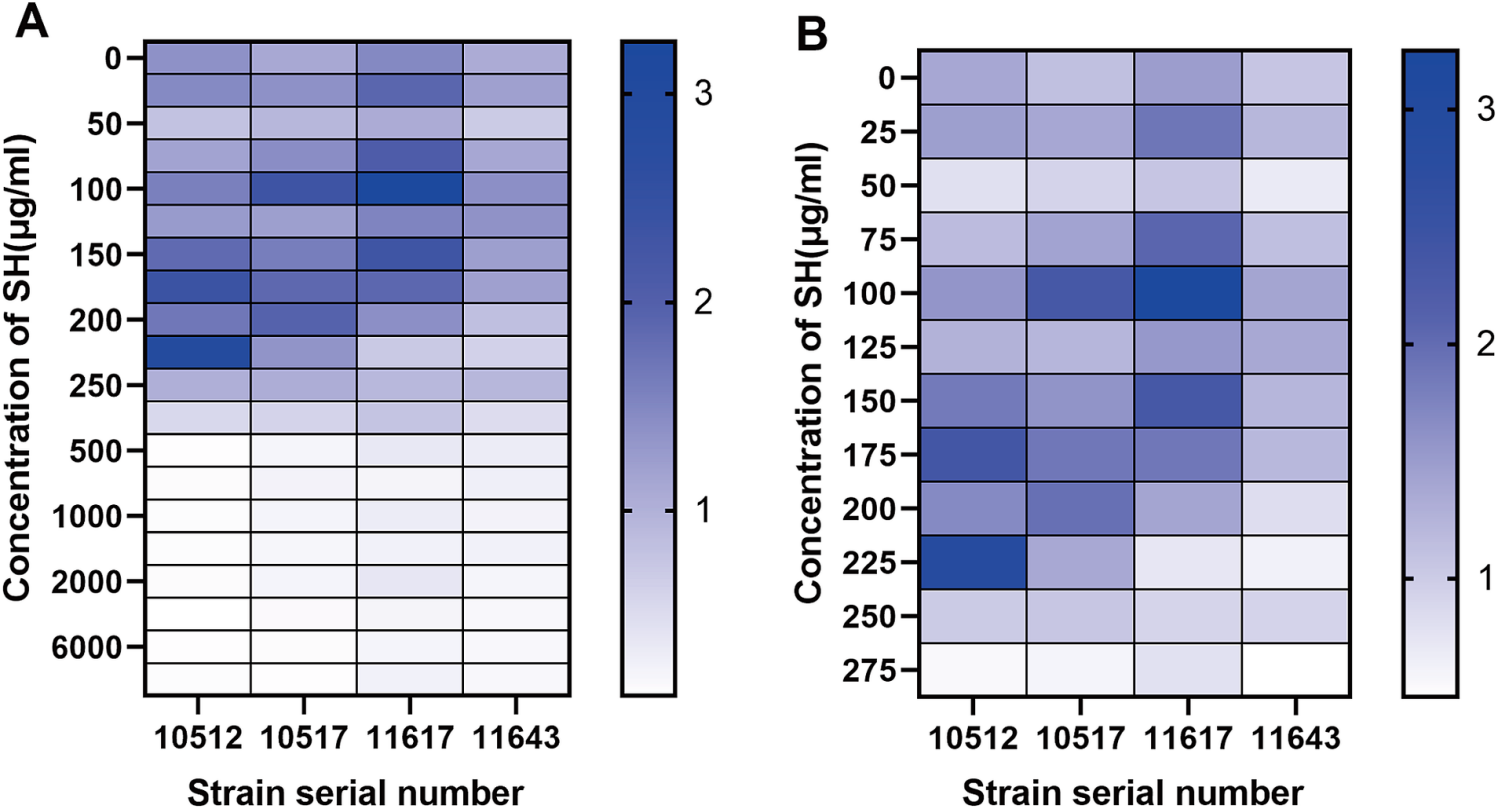
Thermal efficacy diagram of bioflim. **(A)** Different concentration of SH. **(B)** Substatic concentration of SH.

Crystal violet staining revealed dose-dependent biofilm inhibition by SH (≥250 μg/ml, *p* < 0.001 vs. untreated control). Notably, sub-MIC concentrations (25–125 μg/ml) transiently enhanced biofilm formation (Strain 10,512 and Strain 11,617 *p* < 0.05), followed by suppression at ≥250 μg/ml (Figures 5–7). This biphasic response suggests SH may initially promote bacterial attachment at low doses before exerting inhibitory effects.

### 3.6 Biofilm architecture disruption

**Figure 8:**
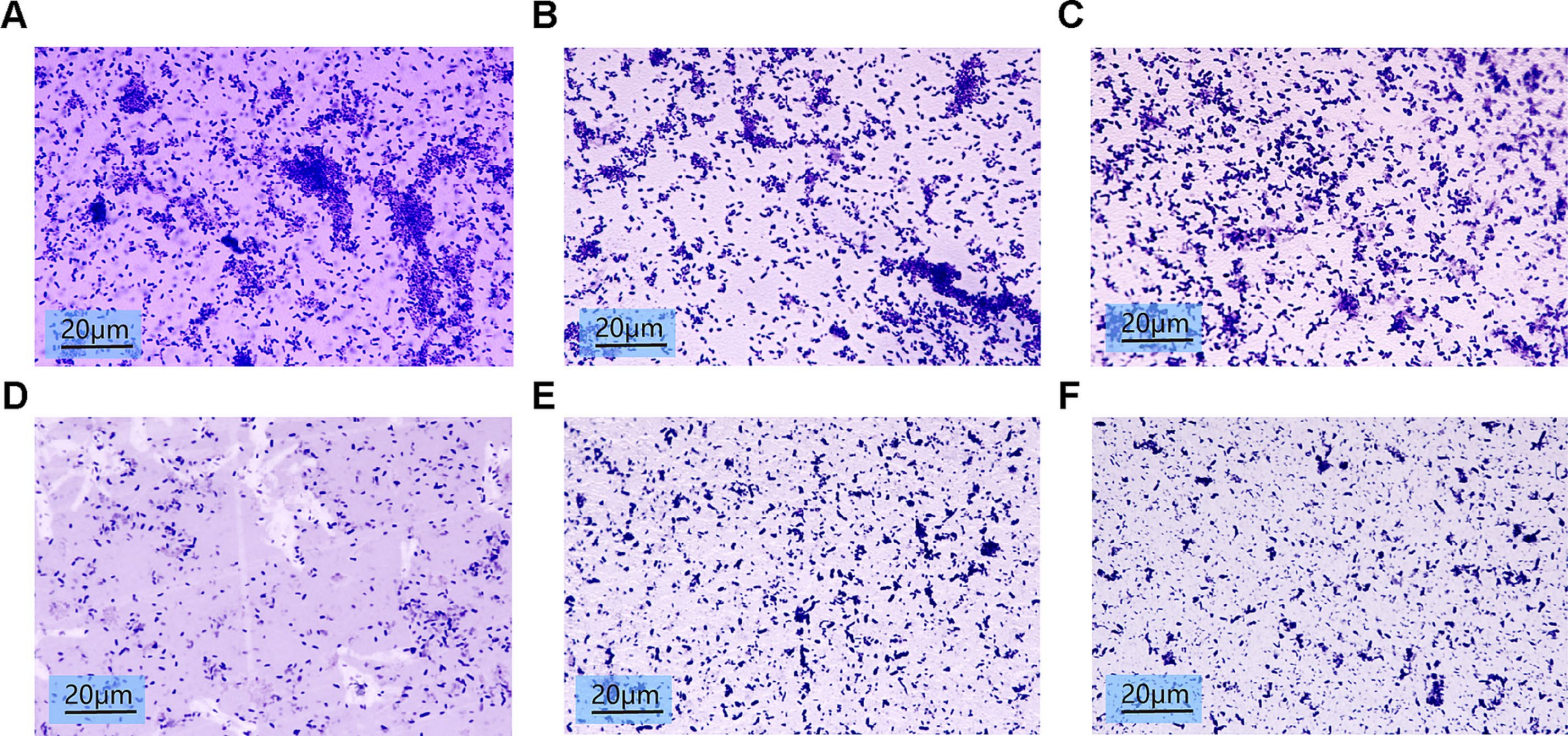
Biofilm images of crystalline violet stained 10*100x light microscopy after treatment with different concentrations of Sodium houttuyfonate. **(A)** 0 μg/ml SH. **(B)** 250 μg/ml SH. **(C)** 500 μg/ml SH. **(D)** 1,000 μg/ml SH. **(E)** 2,000 μg/ml SH. **(F)** 4,000 μg/ml SH.

Microscopic analysis of crystal violet-stained biofilms showed reduced bacterial density and structural loosening in SH-treated groups (Figure 8). Higher SH concentrations (>500 μg/ml) correlated with diminished biofilm viscosity and dispersal of bacterial clusters.

### 3.7 Motility suppression

**Figure 9:**
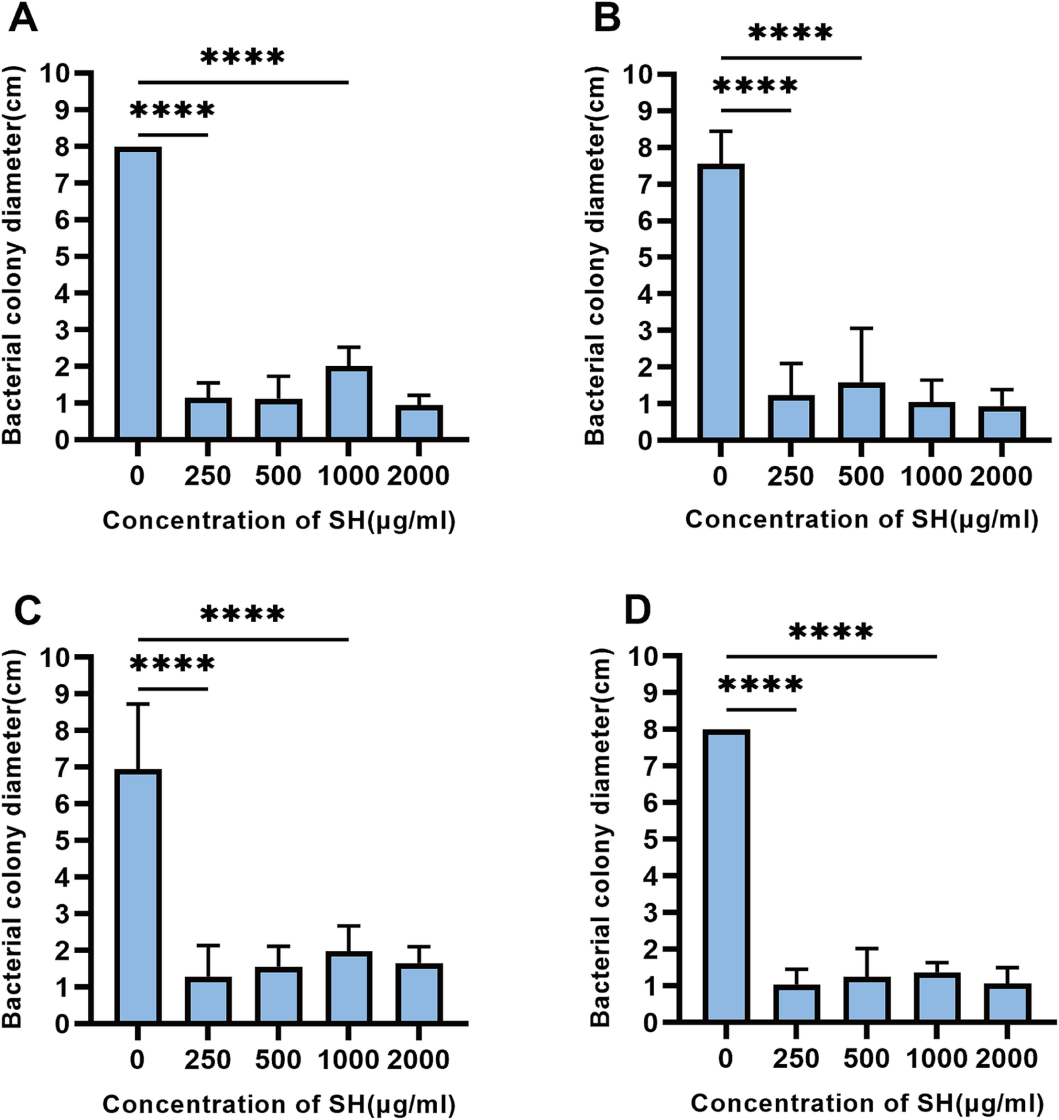
Sodium houttuyfonate can inhibit the swimming ability of *Pseudomonas aeruginosa*. A ~ D denote the size of the diameter of colonies grown by experimental strains in plates containing different concentrations of Sodium houttuyfonate. **(A)** 10,512. **(B)** 10,517. **(C)** 11,617. **(D)** 11,643. Where ns denotes no statistical significance, * denotes 0.05 ≤ *p* < 0.1, ** denotes *p* < 0.05, *** denotes *p* < 0.01, and **** denotes *p* < 0.001.Error bars denote SD.

**Figure 10:**
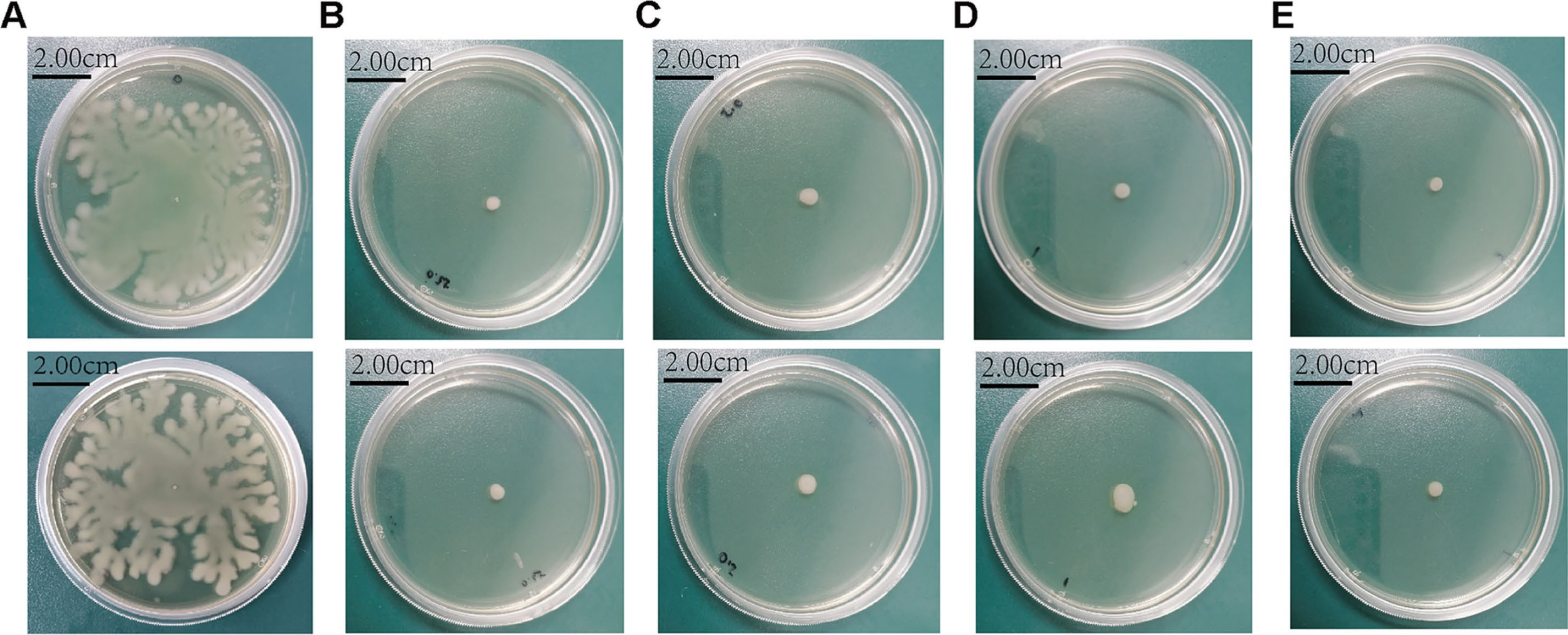
The morphology of colonies grown in plates with different concentrations of Sodium houttuyfonate. **(A)** 0 μg/ml SH. **(B)** 250 μg/ml SH. **(C)** 500 μg/ml SH. **(D)** 1,000 μg/ml SH. **(E)** 2,000 μg/ml SH.

SH significantly impaired swimming motility at ≥250 μg/ml, as evidenced by reduced colony diameters on soft agar (*p* < 0.01 vs. control; Figures 9, 10).

### 3.8 Gene expression profiling

**Figure 11:**
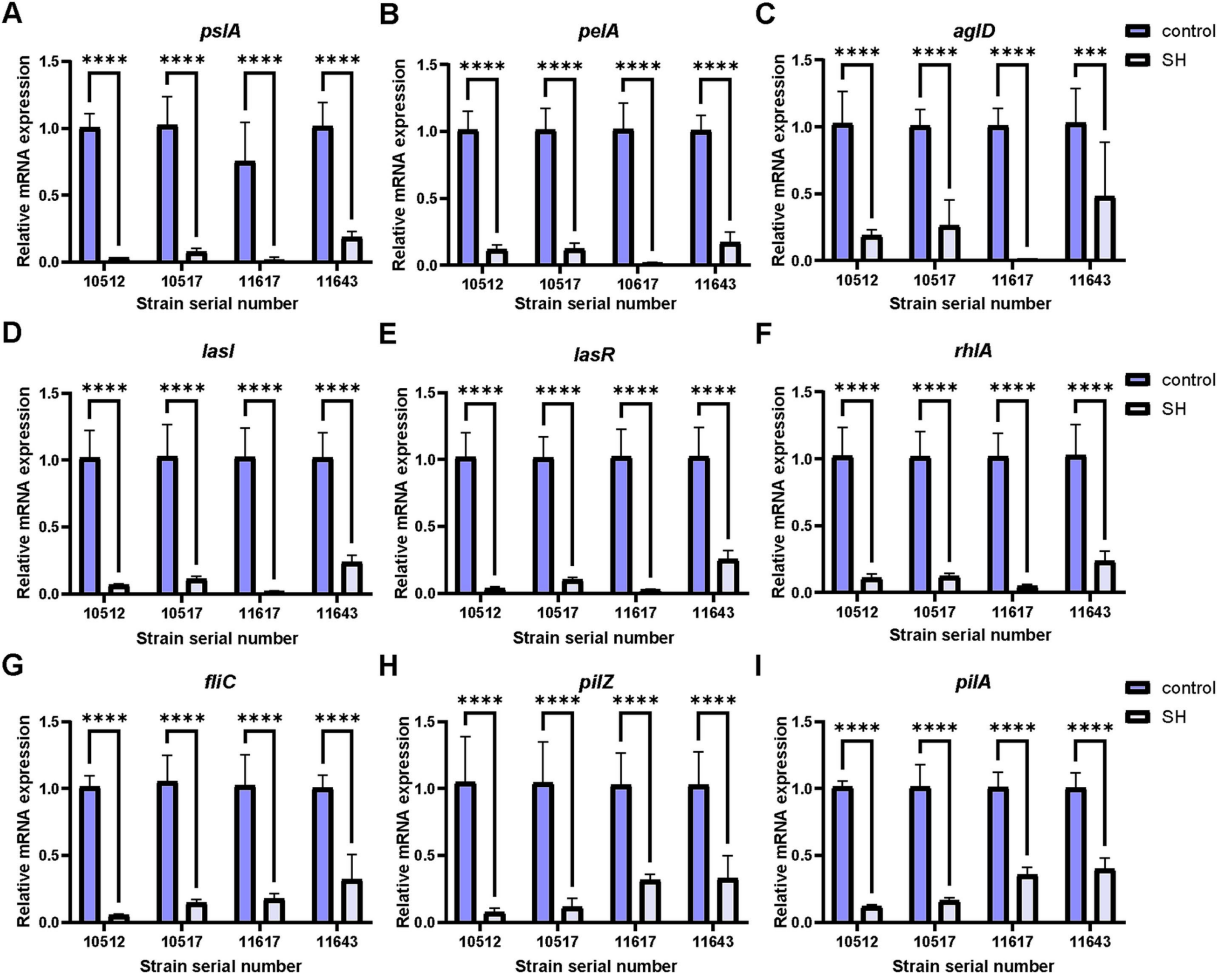
Expression of genes related to biofilm and motor flagellum inhibition by sodium houttuyfonate. A ~ F are genes related to biofilm expression, and G ~ I are genes related to motor flagellum expression. **(A)** *pslA*; **(B)** *pelA*; **(C)** *aglD*; **(D)** *lasI*; **(E)** *lasR*; **(F)** *rhlA*; **(G)** *fliC*; **(H)** *pilZ*; **(I)** *pilA*. Where ns denotes no statistical significance, * denotes 0.05 ≤ *p* < 0.1, ** denotes *p* < 0.05, *** denotes *p* < 0.01, and **** denotes *p* < 0.001.Error bars denote SD.

**Figure 12:**
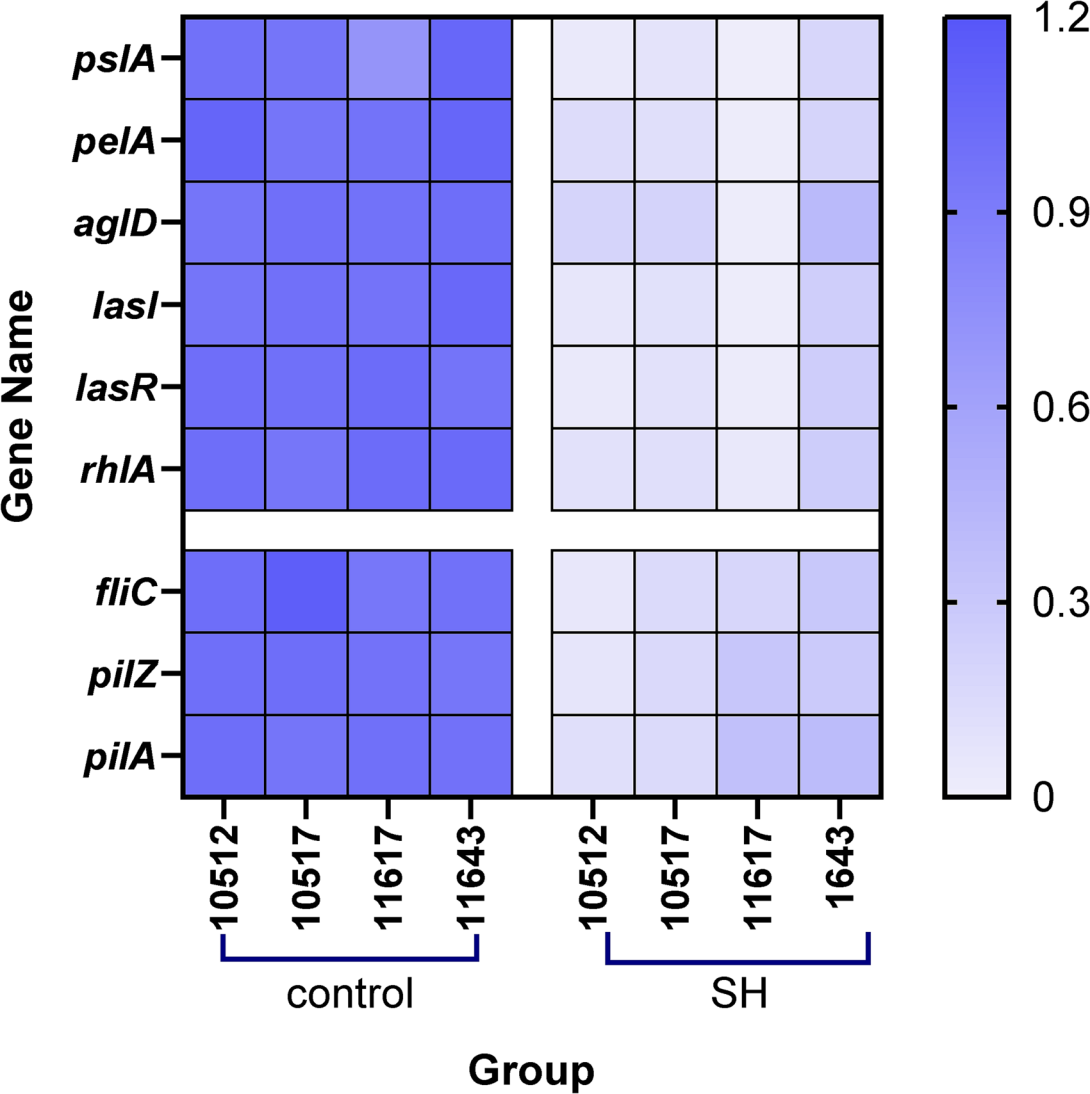
Heatmap of relative gene expression.

qRT-PCR analysis indicated SH downregulated biofilm-related genes (*pslA*, *pelA*, *algD*, *lasI*, *lasR*, *rhlA*) and motility-associated genes (*fliC, pilA*, *pilZ*) (*p* < 0.01; Figures 11, 12). These findings align with observed phenotypic changes in biofilm and motility assays.

## 4 Discussion

### 4.1 Key findings summary

This study demonstrates that Sodium houttuyfonate synergizes with Meropenem to combat heteroresistant *Pseudomonas aeruginosa* by targeting both planktonic and biofilm-associated virulence. Our results highlight three critical advances:

- Synergistic bactericidal activity: SH-MEM combinations (FICI ≤ 0.5) achieved sustained suppression of bacterial regrowth, outperforming monotherapies in time-kill assays.
- Dual-phase biofilm modulation: SH exhibited a biphasic effect—sub-MIC concentrations (25–125 μg/ml) transiently enhanced biofilm formation, while ≥250 μg/ml suppressed it through downregulation of matrix genes (*pslA, pelA*) and quorum sensing regulators (*lasI*/*lasR*).
- Multi-target gene suppression: SH disrupted motility via *fliC* (flagellin) and *pilA*/*pilZ* (type IV pili), while concurrently inhibiting virulence regulators (*rhlA*, *algD*) linked to immune evasion.

### 4.2 Mechanistic insights and literature comparison

#### 4.2.1 Synergy against heteroresistance

The observed synergy between Sodium houttuyfonate and Meropenem addresses heteroresistance, a phenomenon where bacterial subpopulations with varying drug tolerance coexist within a strain. Heteroresistant strains are usually recognised as sensitive strains in automated drug susceptibility testing, which results in patients being infected with that strain, and treatment with conventional antibiotics is often suggestive of poor results (Tam et al., 2005; Sun et al., 2015). Recent studies indicate that heteroresistance to carbapenems in *Pseudomonas aeruginosa* has risen to 58.2% in clinical isolates (Lu et al., 2022), underscoring the urgency for adjuvant therapies. Unlike prior work focusing on SH-levofloxacin synergy (Wang et al., 2002), our study is the first to demonstrate SH-MEM combinatorial efficacy against heteroresistant strains, likely through SH’s disruption of biofilm integrity and MEM’s enhanced penetration. In terms of biofilm disruption, SH downregulates *pslA* (exopolysaccharide synthesis) and *pelA* (Pel polysaccharide deacetylation), destabilizing biofilm architecture and enhancing MEM penetration. This mechanism aligns with studies demonstrating that *pslA* and *pelA* are critical for biofilm structural integrity in *Pseudomonas aeruginosa* (Overhage et al., 2005). Regarding efflux pump modulation, sub-MIC concentrations of SH may transiently inhibit efflux systems (e.g., MexAB-OprM), reducing MEM efflux and increasing intracellular accumulation (Liu et al., 2023). The observed SH-MEM synergy in this study is likely mediated through these dual mechanisms.

#### 4.2.2 Biofilm dynamics and transcriptional regulation

SH exhibits a biphasic biofilm response—low concentrations (25–125 μg/ml) transiently enhance biomass via stress-induced aggregation, while higher doses (≥250 μg/ml) suppress biofilm by 70% (*p* < 0.001). Sub-MIC SH may induce reactive oxygen species (ROS) and upregulating *pelA*-mediated Pel polysaccharide synthesis to temporarily achieve increased biofilm (Liu et al., 2024). At supra-MIC levels, SH disrupts biofilm through transcriptional repression of *pslA* (exopolysaccharides) and *pelA* (deacetylation), while concurrently inhibiting the *lasI/lasR* quorum sensing (QS) system, which governs pyocyanin and elastase production (Ambreetha and Singh, 2023). SH’s dual-phase action positions it as a “biofilm modulator,” offering precision in targeting biofilm-embedded pathogens.

In a previous report, the *aglD* gene could encode GDP mannitol dehydrogenase, whose transcriptional activation was shown to be required for alginate production (Deretic et al., 1987). The expression of *aglD* showed a positive correlation with biofilm formation, and our study is the same as the previous study by Wu et al. (2014), which showed that Sodium houttuyfonate reduces alginate production by decreasing the expression of *aglD*, which ultimately leads to the inhibition of biofilm formation. Rhamnose lipids have been shown to cause lysis of immune cells in previous reports, whereas in animal experiments, inactivation of the *rhlA* gene is thought to prevent the production of rhamnose lipids, thereby depriving *Pseudomonas aeruginosa* of the “biofilm shielding effect” of rhamnose and allowing it to be cleared by the immune system (Soberon-Chavez et al., 2005; Van Gennip et al., 2009). In our study, the expression of *rhlA* gene in *Pseudomonas aeruginosa* after Sodium houttuyfonate treatment was significantly decreased which in turn led to a decrease in the production of rhamnoglycan lipids and ultimately counted the de-biofilm shielding effect.

*Pseudomonas aeruginosa* has three widely reported population sensing systems, the more important of which is the *lasI*/*lasR* system, or Las system, in which *lasR* is a transcriptional regulator and *lasI* is a synthase protein (Stintzi et al., 1998). *lasI* activates *lasR* into a greening factor by regulating 3O-C12-HSL, one of the most important molecules for inducing *Pseudomonas aeruginosa* population sensing, and interacting with *lasI* to produce biofilm multimers, a process that is involved in the expression of a number of virulence factors that are involved in the pathogenesis of acute infections, including exotoxin A, *lasA* and *lasB* elastases, and alkaline proteases (Kariminik et al., 2017). Our study showed that Sodium houttuyfonate caused inhibition of the expression of the *lasI*/*lasR* system, which implies that SH reduced the population effect and virulence of *Pseudomonas aeruginosa*.

#### 4.2.3 Motility suppression

Previous studies have shown that *Pseudomonas aeruginosa* exhibits swarming, flagellar, and twitching movements. And flagella play an important role in the movement of *Pseudomonas aeruginosa* (Rashid and Kornberg, 2000), among which type IV flagella dependent tic movement is necessary for the formation of surface attached biofilms (Haley et al., 2014). Flagella filaments are formed by the aggregation of a single protein, *fliC* flagellin (Song and Yoon, 2014). Studies have shown that mutations in the flagellin gene *fliC* of *Pseudomonas aeruginosa* can cause bacteria to lose motility but maintain adhesion (Borrero-de Acuna et al., 2015). In 1996, the *pilZ* gene was first proposed to be closely associated with the type IV pili of *Pseudomonas aeruginosa*. It was proposed that *pilZ* can restore the expression of surface pili of *Pseudomonas aeruginosa*, as well as the sensitivity of twitching movement and pili specific phages (Alm et al., 1996). Knocking out the *pilZ* gene in *Pseudomonas aeruginosa* will prevent the assembly of type IV pili on the bacterial surface to inhibit convulsive movement (Qi et al., 2012). The type IV pili of *Pseudomonas aeruginosa* are also related to *pilA*, and the *pilA* pili protein may increase invasiveness to host cells by regulating calcium signaling (Okuda et al., 2013). In animal experiments, mutations in *pilA* can result in the loss of bacterial flagella (Lorenz et al., 2004). Our research suggests that SH may reduce the synthesis of bacterial flagella by reducing the expression of *fliC*, and by reducing the expression of *pilZ* and *pilA* to reduce the synthesis of type IV flagella, ultimately achieving inhibition of bacterial swimming ability. This is consistent with the strong inhibitory effect of SH at lower concentrations in bacterial swimming motility assay.

## 5 Conclusion

This study demonstrates that Sodium houttuyfonate synergizes with Meropenem against heteroresistant *Pseudomonas aeruginosa* through dual mechanisms: (1) SH-MEM combinations (FICI ≤0.5) achieve potent bactericidal activity, suppressing bacterial regrowth for 72 h; (2) SH disrupts biofilm formation and motility by downregulating matrix genes (pslA, pelA) and quorum sensing regulators (*lasI/lasR*), while concurrently inhibiting flagellar (*fliC*) and type IV pili (*pilA*/*pilZ*) expression. Notably, SH exhibits a biphasic biofilm modulation—transient enhancement at sub-MIC (25–125 μg/ml) via stress-induced aggregation, followed by 70% suppression at ≥250 μg/ml (*p* < 0.001). These findings position SH as both a synergistic adjuvant for carbapenem therapies and a standalone anti-biofilm agent. Future studies should validate these findings *in vivo* and explore SH’s interactions with other virulence factors (e.g., lipase, proteas) to provide an optimal direction for the treatment of *Pseudomonas aeruginosa* infection.

## Data Availability

The raw data supporting the conclusions of this article will be made available by the authors, without undue reservation.

## References

[ref1] Alm R. A. Bodero A. J. Free P. D. Mattick J. S. (1996). Identification of a novel gene, pilZ, essential for type 4 fimbrial biogenesis in *Pseudomonas aeruginosa*. J. Bacteriol. 178, 46–53. doi: 10.1128/jb.178.1.46-53.1996, PMID: 8550441 PMC177619

[ref2] Ambreetha S. Singh V. (2023). Genetic and environmental determinants of surface adaptations in *Pseudomonas aeruginosa*. Microbiology 169:1335. doi: 10.1099/mic.0.001335, PMID: 37276014 PMC10333789

[ref3] Borrero-de Acuna J. M. Molinari G. Rohde M. Dammeyer T. Wissing J. Jansch L.. (2015). A periplasmic complex of the nitrite reductase NirS, the chaperone DnaK, and the flagellum protein FliC is essential for flagellum assembly and motility in *Pseudomonas aeruginosa*. J. Bacteriol. 197, 3066–3075. doi: 10.1128/JB.00415-15, PMID: 26170416 PMC4560289

[ref4] Bubonja-Sonje M. Matovina M. Skrobonja I. Bedenic B. Abram M. (2015). Mechanisms of Carbapenem resistance in multidrug-resistant clinical isolates of *Pseudomonas aeruginosa* from a Croatian hospital. Microb. Drug Resist. 21, 261–269. doi: 10.1089/mdr.2014.0172, PMID: 25565041

[ref5] Buehrle D. J. Shields R. K. Clarke L. G. Potoski B. A. Clancy C. J. Nguyen M. H. (2017). Carbapenem-resistant *Pseudomonas aeruginosa* bacteremia: risk factors for mortality and microbiologic treatment failure. Antimicrob. Agents Chemother. 61:16. doi: 10.1128/AAC.01243-16, PMID: 27821456 PMC5192105

[ref6] Deretic V. Gill J. F. Chakrabarty A. M. (1987). *Pseudomonas aeruginosa* infection in cystic fibrosis: nucleotide sequence and transcriptional regulation of the algD gene. Nucleic Acids Res. 15, 4567–4581. doi: 10.1093/nar/15.11.4567, PMID: 3108855 PMC340880

[ref7] El-Halfawy O. M. Valvano M. A. (2015). Antimicrobial heteroresistance: an emerging field in need of clarity. Clin. Microbiol. Rev. 28, 191–207. doi: 10.1128/CMR.00058-14, PMID: 25567227 PMC4284305

[ref8] Haley C. L. Kruczek C. Qaisar U. Colmer-Hamood J. A. Hamood A. N. (2014). Mucin inhibits *Pseudomonas aeruginosa* biofilm formation by significantly enhancing twitching motility. Can. J. Microbiol. 60, 155–166. doi: 10.1139/cjm-2013-0570, PMID: 24588389 PMC4199588

[ref9] Haney E. F. Trimble M. J. Hancock R. E. W. (2021). Microtiter plate assays to assess antibiofilm activity against bacteria. Nat. Protoc. 16, 2615–2632. doi: 10.1038/s41596-021-00515-3, PMID: 33911258

[ref10] Kariminik A. Baseri-Salehi M. Kheirkhah B. (2017). *Pseudomonas aeruginosa* quorum sensing modulates immune responses: An updated review article. Immunol. Lett. 190, 1–6. doi: 10.1016/j.imlet.2017.07.002, PMID: 28698104

[ref11] Kumar M. Prasad S. K. Hemalatha S. (2014). A current update on the phytopharmacological aspects of *Houttuynia cordata* Thunb. Pharmacogn. Rev. 8, 22–35. doi: 10.4103/0973-7847.125525, PMID: 24600193 PMC3931198

[ref12] Laldinsangi C. (2022). The therapeutic potential of *Houttuynia cordata*: a current review. Heliyon 8:e10386. doi: 10.1016/j.heliyon.2022.e10386, PMID: 36061012 PMC9433674

[ref13] Liu Q. Tang Y. Jiang S. Yu X. Zhu H. Xie X.. (2024). Mechanisms of action of berberine hydrochloride in planktonic cells and biofilms of *Pseudomonas aeruginosa*. Microb. Pathog. 193:106774. doi: 10.1016/j.micpath.2024.106774, PMID: 38969184

[ref14] Liu Y. Zhu R. Liu X. Li D. Guo M. Fei B.. (2023). Effect of piperine on the inhibitory potential of MexAB-OprM efflux pump and imipenem resistance in carbapenem-resistant *Pseudomonas aeruginosa*. Microb. Pathog. 185:106397. doi: 10.1016/j.micpath.2023.106397, PMID: 37852553

[ref15] Lorenz E. Chemotti D. C. Vandal K. Tessier P. A. (2004). Toll-like receptor 2 represses nonpilus adhesin-induced signaling in acute infections with the *Pseudomonas aeruginosa* pilA mutant. Infect. Immun. 72, 4561–4569. doi: 10.1128/IAI.72.8.4561-4569.2004, PMID: 15271916 PMC470691

[ref16] Lu Y. Liu Y. Zhou C. Liu Y. Long Y. Lin D.. (2022). Quorum sensing regulates heteroresistance in *Pseudomonas aeruginosa*. Front. Microbiol. 13:1017707. doi: 10.3389/fmicb.2022.1017707, PMID: 36386621 PMC9650436

[ref17] Masihzadeh S. Amin M. Farshadzadeh Z. (2023). In vitro and in vivo antibiofilm activity of the synthetic antimicrobial peptide WLBU2 against multiple drug resistant *Pseudomonas aeruginosa* strains. BMC Microbiol. 23:131. doi: 10.1186/s12866-023-02886-x, PMID: 37183241 PMC10184367

[ref18] Okuda J. Hayashi N. Arakawa M. Minagawa S. Gotoh N. (2013). Type IV pilus protein PilA of *Pseudomonas aeruginosa* modulates calcium signaling through binding the calcium-modulating cyclophilin ligand. J. Infect. Chemother. 19, 653–664. doi: 10.1007/s10156-012-0536-y, PMID: 23266901

[ref19] Overhage J. Schemionek M. Webb J. S. Rehm B. H. (2005). Expression of the psl operon in *Pseudomonas aeruginosa* PAO1 biofilms: PslA performs an essential function in biofilm formation. Appl. Environ. Microbiol. 71, 4407–4413. doi: 10.1128/AEM.71.8.4407-4413.2005, PMID: 16085831 PMC1183271

[ref20] Parvekar P. Palaskar J. Metgud S. Maria R. Dutta S. (2020). The minimum inhibitory concentration (MIC) and minimum bactericidal concentration (MBC) of silver nanoparticles against *Staphylococcus aureus*. Biomater. Investig. Dent. 7, 105–109. doi: 10.1080/26415275.2020.1796674, PMID: 32939454 PMC7470068

[ref21] Pulmonary Infection Assembly of Chinese Thoracic (2022). Chinese expert consensus on the management of lower respiratory tract infections of *Pseudomonas aeruginosa* in adults (2022). Zhonghua Jie He He Hu Xi Za Zhi 45, 739–752. doi: 10.3760/cma.j.cn112147-20220407-00290, PMID: 35927044

[ref22] Qi Y. Xu L. Dong X. Yau Y. H. Ho C. L. Koh S. L.. (2012). Functional divergence of FimX in PilZ binding and type IV pilus regulation. J. Bacteriol. 194, 5922–5931. doi: 10.1128/JB.00767-12, PMID: 22942245 PMC3486077

[ref23] Qu L. She P. Wang Y. Liu F. Zhang D. Chen L.. (2016). Effects of norspermidine on *Pseudomonas aeruginosa* biofilm formation and eradication. Microbiology 5, 402–412. doi: 10.1002/mbo3.338, PMID: 26817804 PMC4905993

[ref24] Rashid M. H. Kornberg A. (2000). Inorganic polyphosphate is needed for swimming, swarming, and twitching motilities of *Pseudomonas aeruginosa*. Proc. Natl. Acad. Sci. USA 97, 4885–4890. doi: 10.1073/pnas.060030097, PMID: 10758151 PMC18327

[ref25] Rogers A. T. Bullard K. R. Dod A. C. Wang Y. (2022). Bacterial growth curve measurements with a multimode microplate reader. Bio. Protoc. 12:e4410. doi: 10.21769/BioProtoc.4410, PMID: 35800461 PMC9090524

[ref26] Soberon-Chavez G. Lepine F. Deziel E. (2005). Production of rhamnolipids by *Pseudomonas aeruginosa*. Appl. Microbiol. Biotechnol. 68, 718–725. doi: 10.1007/s00253-005-0150-3, PMID: 16160828

[ref27] Song W. S. Yoon S. I. (2014). Crystal structure of FliC flagellin from Pseudomonas aeruginosa and its implication in TLR5 binding and formation of the flagellar filament. Biochem. Biophys. Res. Commun. 444, 109–115. doi: 10.1016/j.bbrc.2014.01.008, PMID: 24434155

[ref28] Stintzi A. Evans K. Meyer J. M. Poole K. (1998). Quorum-sensing and siderophore biosynthesis in *Pseudomonas aeruginosa*: lasR/lasI mutants exhibit reduced pyoverdine biosynthesis. FEMS Microbiol. Lett. 166, 341–345. doi: 10.1111/j.1574-6968.1998.tb13910.x, PMID: 9770291

[ref29] Sun J. D. Huang S. F. Yang S. S. Pu S. L. Zhang C. M. Zhang L. P. (2015). Impact of carbapenem heteroresistance among clinical isolates of invasive *Escherichia coli* in Chongqing, southwestern China. Clin. Microbiol. Infect. 21:469.e1. doi: 10.1016/j.cmi.2014.12.013, PMID: 25649300

[ref30] Tam V. H. Schilling A. N. Neshat S. Poole K. Melnick D. A. Coyle E. A. (2005). Optimization of meropenem minimum concentration/MIC ratio to suppress in vitro resistance of *Pseudomonas aeruginosa*. Antimicrob. Agents Chemother. 49, 4920–4927. doi: 10.1128/AAC.49.12.4920-4927.2005, PMID: 16304153 PMC1315965

[ref31] Van Gennip M. Christensen L. D. Alhede M. Phipps R. Jensen P. O. Christophersen L.. (2009). Inactivation of the rhlA gene in *Pseudomonas aeruginosa* prevents rhamnolipid production, disabling the protection against polymorphonuclear leukocytes. APMIS 117, 537–546. doi: 10.1111/j.1600-0463.2009.02466.x, PMID: 19594494 PMC2997331

[ref32] Wang D. Yu Q. Eikstadt P. Hammond D. Feng Y. Chen N. (2002). Studies on adjuvanticity of sodium houttuyfonate and its mechanism. Int. Immunopharmacol. 2, 1411–1418. doi: 10.1016/S1567-5769(02)00060-7, PMID: 12400871

[ref33] Wu D. Q. Cheng H. Duan Q. Huang W. (2015). Sodium houttuyfonate inhibits biofilm formation and alginate biosynthesis-associated gene expression in a clinical strain of *Pseudomonas aeruginosa in vitro*. Exp. Ther. Med. 10, 753–758. doi: 10.3892/etm.2015.2562, PMID: 26622388 PMC4509011

[ref34] Wu Z. Deng X. Hu Q. Xiao X. Jiang J. Ma X.. (2021). *Houttuynia cordata* Thunb: an Ethnopharmacological review. Front. Pharmacol. 12:714694. doi: 10.3389/fphar.2021.714694, PMID: 34539401 PMC8440972

[ref35] Wu D. Huang W. Duan Q. Li F. Cheng H. (2014). Sodium houttuyfonate affects production of N-acyl homoserine lactone and quorum sensing-regulated genes expression in *Pseudomonas aeruginosa*. Front. Microbiol. 5:635. doi: 10.3389/fmicb.2014.00635, PMID: 25505457 PMC4244979

[ref36] Yin Y. Zhao C. Li H. Jin L. Wang Q. Wang R.. (2021). Clinical and microbiological characteristics of adults with hospital-acquired pneumonia: a 10-year prospective observational study in China. Eur. J. Clin. Microbiol. Infect. Dis. 40, 683–690. doi: 10.1007/s10096-020-04046-9, PMID: 33029764 PMC7540435

