# Supplementary material for: Antibacterial activity and mechanism of Sodium houttuyfonate against heteroresistant Pseudomonas aeruginosa
Source: Front Microbiol. 2025 Mar 6;16:1523182. doi: 10.3389/fmicb.2025.1523182 (PMC11922930; doi:10.3389/fmicb.2025.1523182)
Supplement: Supplementary file 1 [file Table_1.docx]

**Table S1.** Sequences of real-time fluorescence quantitative PCR primers and their related properties.

| Gene | Primer sequences (5’→3′) | Tm(℃) | GC% | Product size(bp) | effect |
| --- | --- | --- | --- | --- | --- |
| *16SrRNA* | Forward: AACGCGAAGAACCTTACC | 54.99 | 50.00 | 149 |  |
|  | Reverse: AAGGGTTGCGCTCGTTAC | 58.04 | 55.56 |  |  |
| *pslA* | Forward: CGGTCAGCGAATACAGCTC | 58.42 | 57.89 | 103 | Biofilm |
|  | Reverse: TTGATCTTGTGCAGGGTGTC | 58.10 | 50.00 |  |  |
| *pelA* | Forward: GACGTGCAGCGATCCAGTTGT | 63.52 | 57.14 | 159 |  |
|  | Reverse: GCCTGCTCGAATACCTCGGTTATC | 63.14 | 54.17 |  |  |
| *aglD* | Forward: TGATCTGCCAGGACCACAAGC | 63.79 | 61.90 | 186 |  |
|  | Reverse: CGCATCAACGAACCGAGCATC | 57.60 | 57.60 |  |  |
| *lasI* | Forward: ACCGTAGGCGTGGAGAAGATGA | 60.11 | 45.00 | 247 |  |
|  | Reverse: GCGATCTGGGTCTTGGCATTGA | 59.93 | 55.00 |  |  |
| *lasR* | Forward: TCGGTTATCTGCAACTGCTC | 57.99 | 50.00 | 115 |  |
|  | Reverse: GACCCAAATTAACGGCCATA | 55.79 | 45.00 |  |  |
| *rhlA* | Forward: CTCGGCGGTGGTGTATTCGT | 62.55 | 60.00 | 175 |  |
|  | Reverse: AGCCAGCAACCATCAGCACAT | 62.96 | 52.38 |  |  |
| *pilZ* | Forward: CGTCGCCGAGCTTGTAGTTCTT | 63.13 | 54.55 | 115 | Flagellar movement |
|  | Reverse: GGCATCCTGTCCTTGACCATCA | 62.34 | 54.55 |  |  |
| *pilA* | Forward: GCGACAGCGACTCTTCAACAGT | 63.32 | 54.55 | 123 |  |
|  | Reverse: TCATCGGTATCCTGGCGGCAAT | 64.17 | 54.55 |  |  |
| *fliC* | Forward: CGACAAGGGTGTACTGACCA | 59.32 | 55.00 | 100 |  |
|  | Reverse: GACCTTCACTGCGACCTGAC | 60.67 | 60.00 |  |  |
